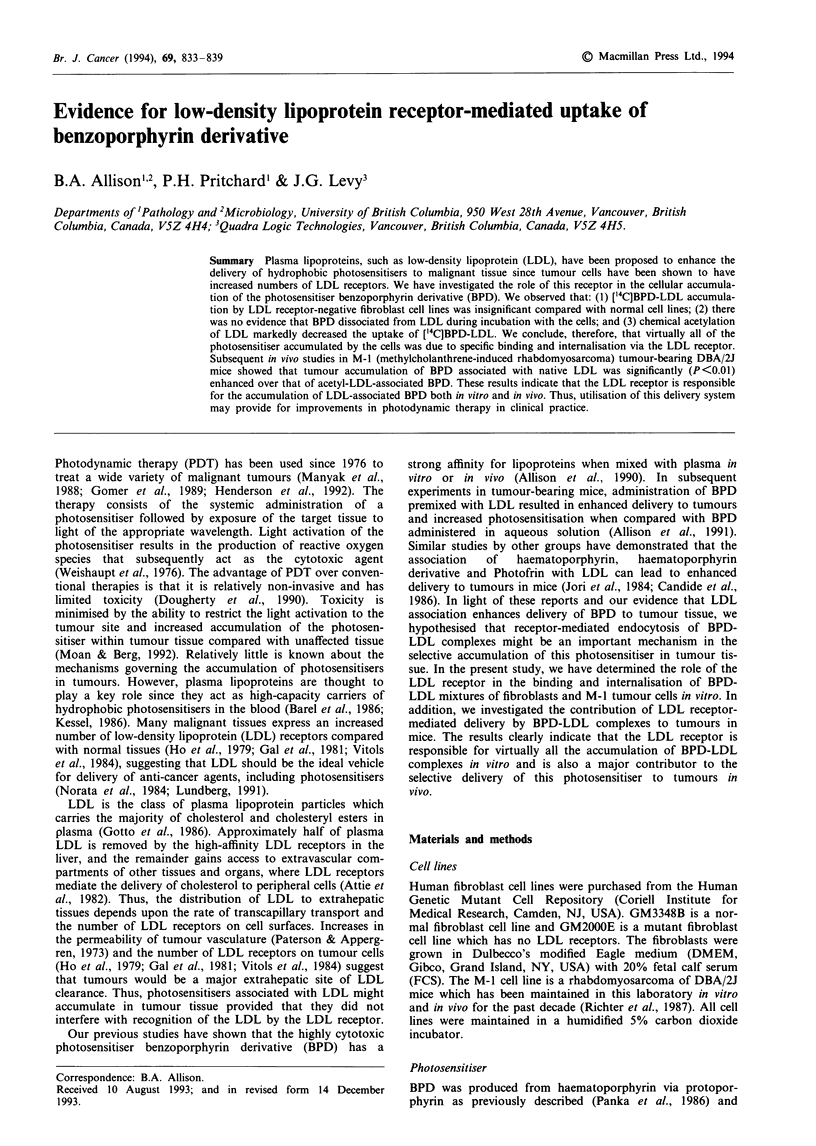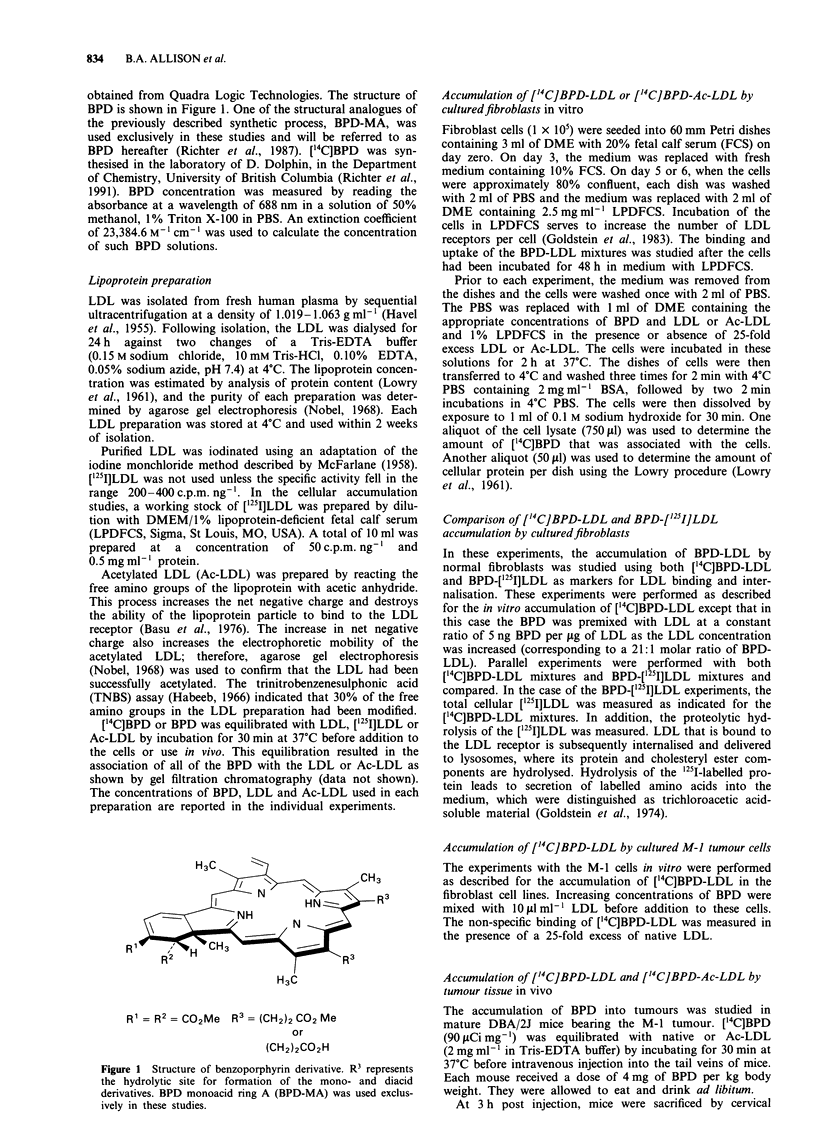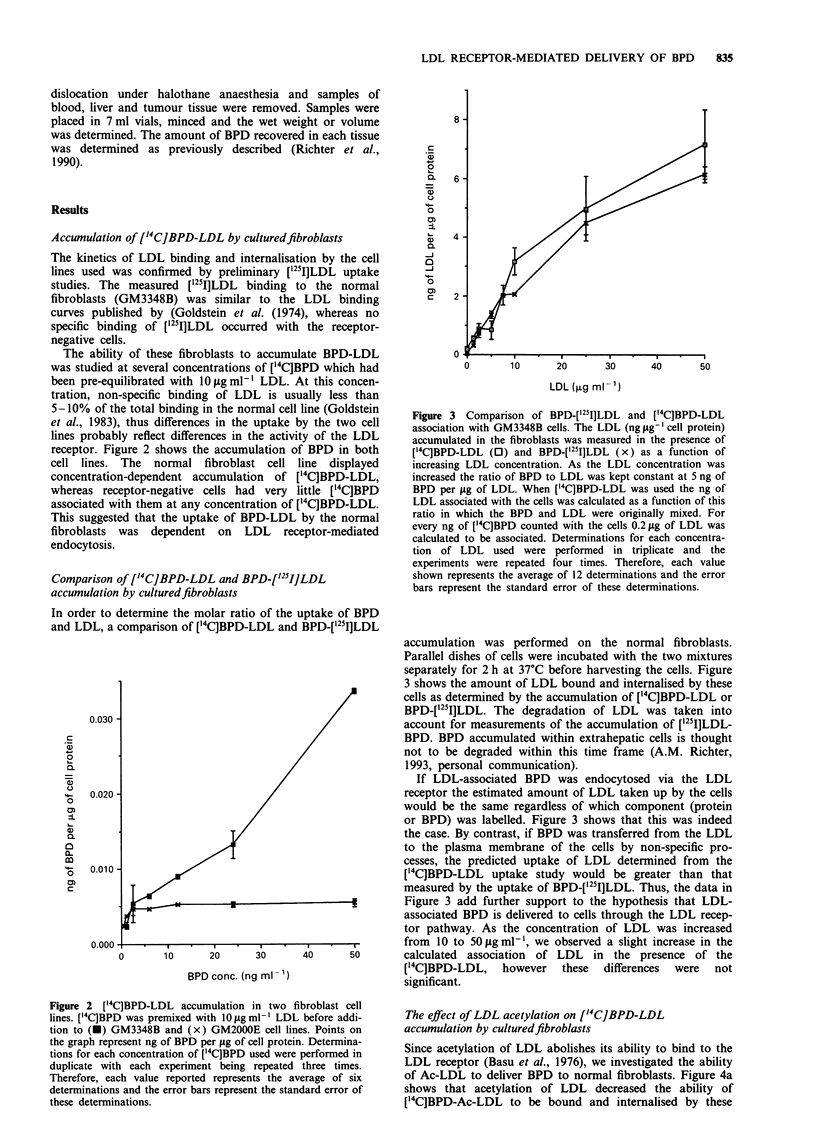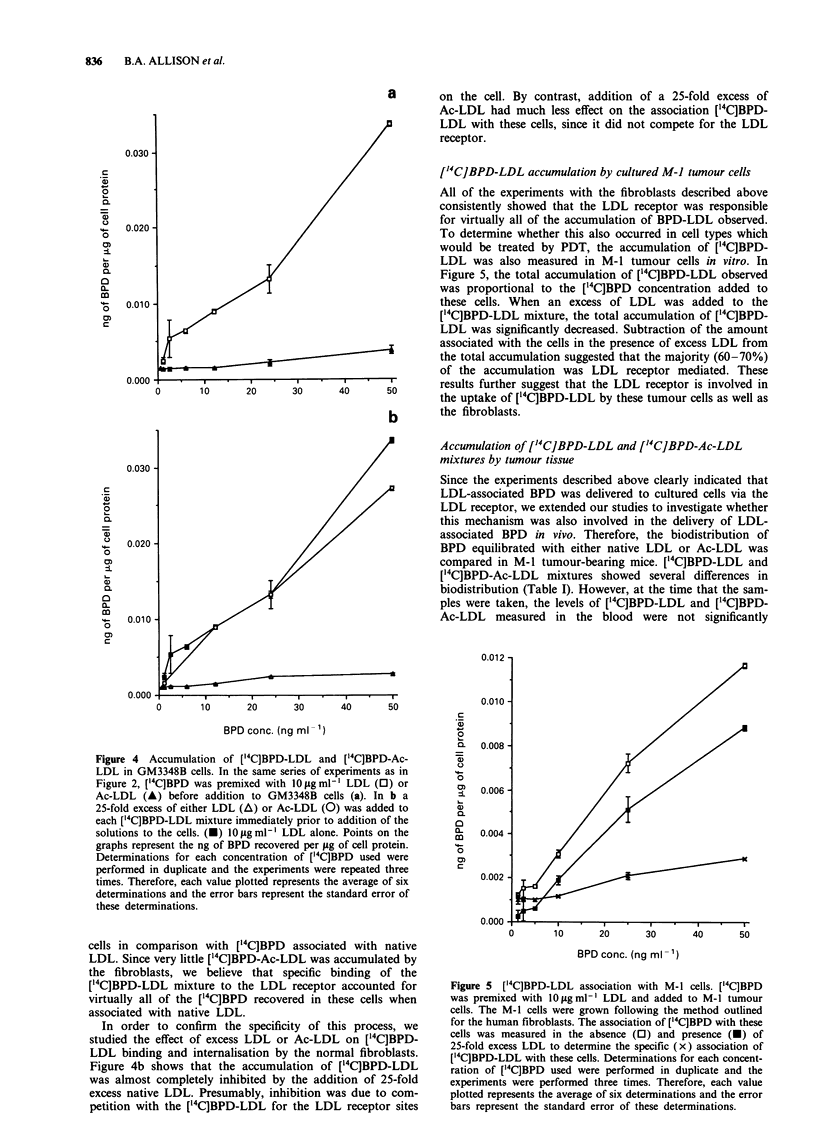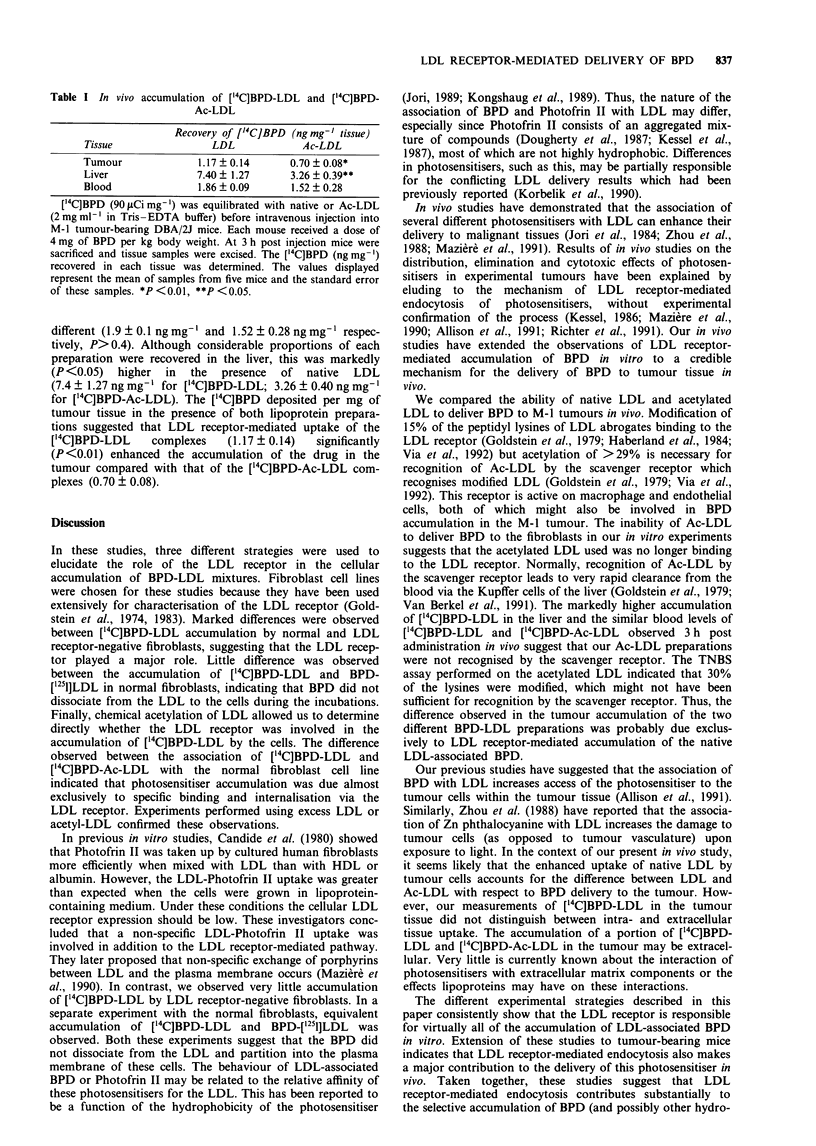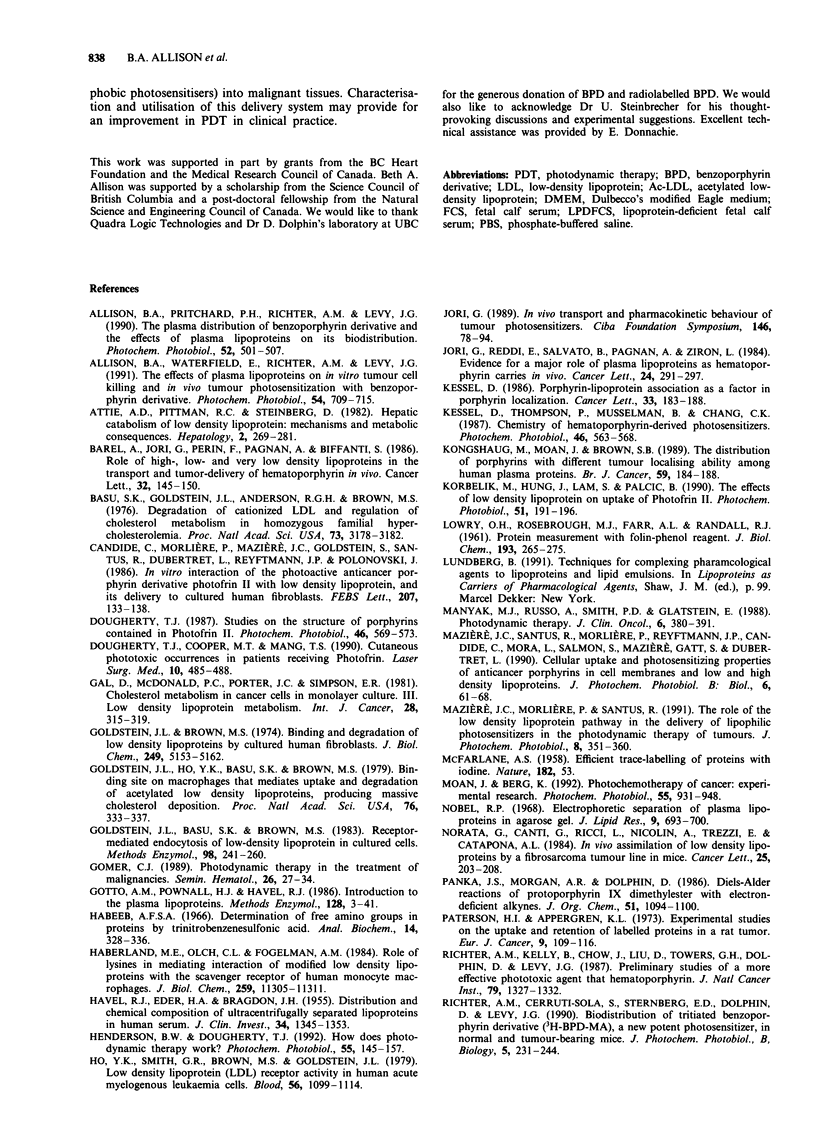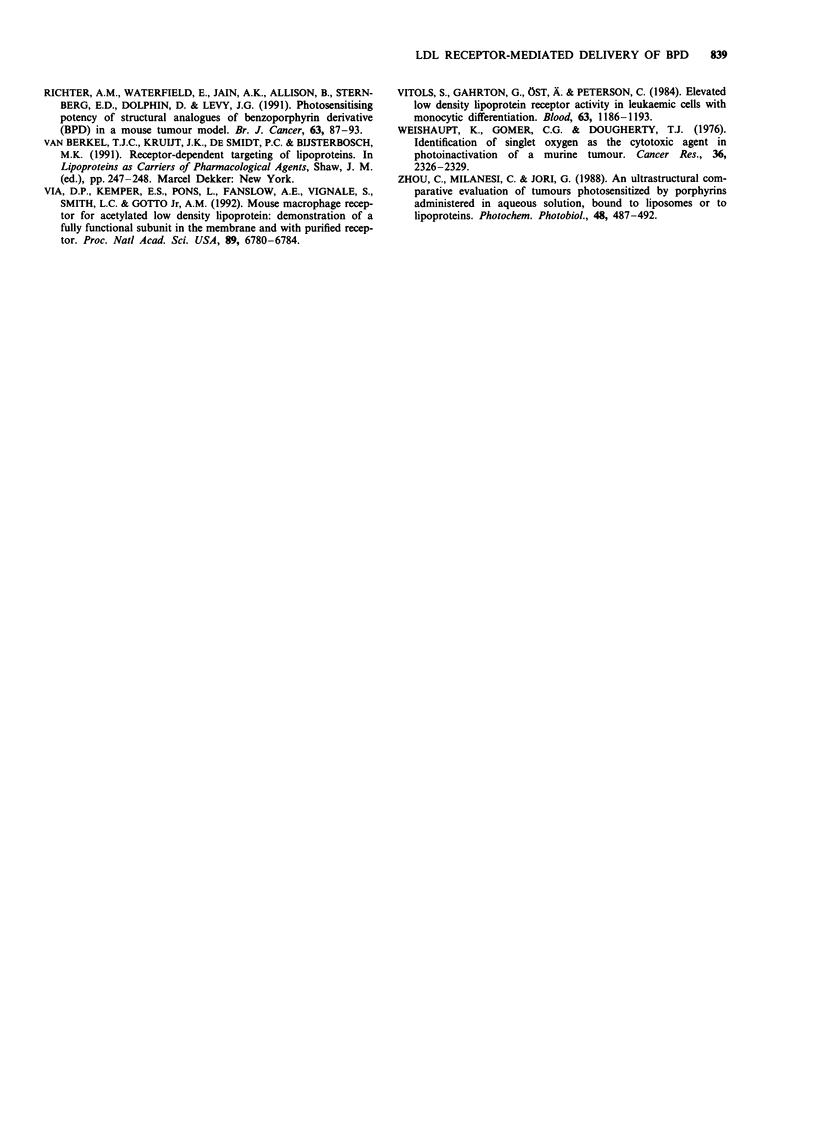# Evidence for low-density lipoprotein receptor-mediated uptake of benzoporphyrin derivative.

**DOI:** 10.1038/bjc.1994.162

**Published:** 1994-05

**Authors:** B. A. Allison, P. H. Pritchard, J. G. Levy

**Affiliations:** Department of Pathology, University of British Columbia, Vancouver, Canada.

## Abstract

Plasma lipoproteins, such as low-density lipoprotein (LDL), have been proposed to enhance the delivery of hydrophobic photosensitisers to malignant tissue since tumour cells have been shown to have increased numbers of LDL receptors. We have investigated the role of this receptor in the cellular accumulation of the photosensitiser benzoporphyrin derivative (BPD). We observed that: (1) [14C]BPD-LDL accumulation by LDL receptor-negative fibroblast cell lines was insignificant compared with normal cell lines; (2) there was no evidence that BPD dissociated from LDL during incubation with the cells; and (3) chemical acetylation of LDL markedly decreased the uptake of [14C]BPD-LDL. We conclude, therefore, that virtually all of the photosensitiser accumulated by the cells was due to specific binding and internalisation via the LDL receptor. Subsequent in vivo studies in M-1 (methylcholanthrene-induced rhabdomyosarcoma) tumour-bearing DBA/2J mice showed that tumour accumulation of BPD associated with native LDL was significantly (P < 0.01) enhanced over that of acetyl-LDL-associated BPD. These results indicate that the LDL receptor is responsible for the accumulation of LDL-associated BPD both in vitro and in vivo. Thus, utilisation of this delivery system may provide for improvements in photodynamic therapy in clinical practice.


Br. J. Cancer (1994), 69, 833–839 © Macmillan Press Ltd., 1994

### Evidence for low-density lipoprotein receptor-mediated uptake of benzoporphyrin derivative

B.A. Allison[1,2], P.H. Pritchard[1] & J.G. Levy[3]

*Departments of [1]Pathology and [2]Microbiology, University of British Columbia, 950 West 28th Avenue, Vancouver, British Columbia, Canada, V5Z 4H4; [3]Quadra Logic Technologies, Vancouver, British Columbia, Canada, V5Z 4H5.*

**Summary** Plasma lipoproteins, such as low-density lipoprotein (LDL), have been proposed to enhance the delivery of hydrophobic photosensitisers to malignant tissue since tumour cells have been shown to have increased numbers of LDL receptors. We have investigated the role of this receptor in the cellular accumulation of the photosensitiser benzoporphyrin derivative (BPD). We observed that: (1) [$^{14}$C]BPD-LDL accumulation by LDL receptor-negative fibroblast cell lines was insignificant compared with normal cell lines; (2) there was no evidence that BPD dissociated from LDL during incubation with the cells; and (3) chemical acetylation of LDL markedly decreased the uptake of [$^{14}$C]BPD-LDL. We conclude, therefore, that virtually all of the photosensitiser accumulated by the cells was due to specific binding and internalisation via the LDL receptor. Subsequent *in vivo* studies in M-1 (methylcholanthrene-induced rhabdomyosarcoma) tumour-bearing DBA/2J mice showed that tumour accumulation of BPD associated with native LDL was significantly ($P<0.01$) enhanced over that of acetyl-LDL-associated BPD. These results indicate that the LDL receptor is responsible for the accumulation of LDL-associated BPD both *in vitro* and *in vivo*. Thus, utilisation of this delivery system may provide for improvements in photodynamic therapy in clinical practice.

Photodynamic therapy (PDT) has been used since 1976 to treat a wide variety of malignant tumours (Manyak *et al.*, 1988; Gomer *et al.*, 1989; Henderson *et al.*, 1992). The therapy consists of the systemic administration of a photosensitiser followed by exposure of the target tissue to light of the appropriate wavelength. Light activation of the photosensitiser results in the production of reactive oxygen species that subsequently act as the cytotoxic agent (Weishaupt *et al.*, 1976). The advantage of PDT over conventional therapies is that it is relatively non-invasive and has limited toxicity (Dougherty *et al.*, 1990). Toxicity is minimised by the ability to restrict the light activation to the tumour site and increased accumulation of the photosensitiser within tumour tissue compared with unaffected tissue (Moan & Berg, 1992). Relatively little is known about the mechanisms governing the accumulation of photosensitisers in tumours. However, plasma lipoproteins are thought to play a key role since they act as high-capacity carriers of hydrophobic photosensitisers in the blood (Barel *et al.*, 1986; Kessel, 1986). Many malignant tissues express an increased number of low-density lipoprotein (LDL) receptors compared with normal tissues (Ho *et al.*, 1979; Gal *et al.*, 1981; Vitols *et al.*, 1984), suggesting that LDL should be the ideal vehicle for delivery of anti-cancer agents, including photosensitisers (Norata *et al.*, 1984; Lundberg, 1991).

LDL is the class of plasma lipoprotein particles which carries the majority of cholesterol and cholesteryl esters in plasma (Gotto *et al.*, 1986). Approximately half of plasma LDL is removed by the high-affinity LDL receptors in the liver, and the remainder gains access to extravascular compartments of other tissues and organs, where LDL receptors mediate the delivery of cholesterol to peripheral cells (Attie *et al.*, 1982). Thus, the distribution of LDL to extrahepatic tissues depends upon the rate of transcapillary transport and the number of LDL receptors on cell surfaces. Increases in the permeability of tumour vasculature (Paterson & Appergren, 1973) and the number of LDL receptors on tumour cells (Ho *et al.*, 1979; Gal *et al.*, 1981; Vitols *et al.*, 1984) suggest that tumours would be a major extrahepatic site of LDL clearance. Thus, photosensitisers associated with LDL might accumulate in tumour tissue provided that they did not interfere with recognition of the LDL by the LDL receptor.

Our previous studies have shown that the highly cytotoxic photosensitiser benzoporphyrin derivative (BPD) has a strong affinity for lipoproteins when mixed with plasma *in vitro* or *in vivo* (Allison *et al.*, 1990). In subsequent experiments in tumour-bearing mice, administration of BPD premixed with LDL resulted in enhanced delivery to tumours and increased photosensitisation when compared with BPD administered in aqueous solution (Allison *et al.*, 1991). Similar studies by other groups have demonstrated that the association of haematoporphyrin, haematoporphyrin derivative and Photofrin with LDL can lead to enhanced delivery to tumours in mice (Jori *et al.*, 1984; Candide *et al.*, 1986). In light of these reports and our evidence that LDL association enhances delivery of BPD to tumour tissue, we hypothesised that receptor-mediated endocytosis of BPD-LDL complexes might be an important mechanism in the selective accumulation of this photosensitiser in tumour tissue. In the present study, we have determined the role of the LDL receptor in the binding and internalisation of BPD-LDL mixtures of fibroblasts and M-1 tumour cells *in vitro*. In addition, we investigated the contribution of LDL receptor-mediated delivery by BPD-LDL complexes to tumours in mice. The results clearly indicate that the LDL receptor is responsible for virtually all the accumulation of BPD-LDL complexes *in vitro* and is also a major contributor to the selective delivery of this photosensitiser to tumours *in vivo*.

### Materials and methods

### *Cell lines*

Human fibroblast cell lines were purchased from the Human Genetic Mutant Cell Repository (Coriell Institute for Medical Research, Camden, NJ, USA). GM3348B is a normal fibroblast cell line and GM2000E is a mutant fibroblast cell line which has no LDL receptors. The fibroblasts were grown in Dulbecco's modified Eagle medium (DMEM, Gibco, Grand Island, NY, USA) with 20% fetal calf serum (FCS). The M-1 cell line is a rhabdomyosarcoma of DBA/2J mice which has been maintained in this laboratory *in vitro* and *in vivo* for the past decade (Richter *et al.*, 1987). All cell lines were maintained in a humidified 5% carbon dioxide incubator.

### *Photosensitiser*

BPD was produced from haematoporphyrin via protoporphyrin as previously described (Panka *et al.*, 1986) and

Correspondence: B.A. Allison.
Received 10 August 1993; and in revised form 14 December 1993.

obtained from Quadra Logic Technologies. The structure of BPD is shown in Figure 1. One of the structural analogues of the previously described synthetic process, BPD-MA, was used exclusively in these studies and will be referred to as BPD hereafter (Richter *et al.*, 1987). [$^{14}$C]BPD was synthesised in the laboratory of D. Dolphin, in the Department of Chemistry, University of British Columbia (Richter *et al.*, 1991). BPD concentration was measured by reading the absorbance at a wavelength of 688 nm in a solution of 50% methanol, 1% Triton X-100 in PBS. An extinction coefficient of 23,384.6 $M^{-1}\,cm^{-1}$ was used to calculate the concentration of such BPD solutions.

*Lipoprotein preparation*

LDL was isolated from fresh human plasma by sequential ultracentrifugation at a density of 1.019–1.063 g $ml^{-1}$ (Havel *et al.*, 1955). Following isolation, the LDL was dialysed for 24 h against two changes of a Tris-EDTA buffer (0.15 M sodium chloride, 10 mM Tris-HCl, 0.10% EDTA, 0.05% sodium azide, pH 7.4) at 4°C. The lipoprotein concentration was estimated by analysis of protein content (Lowry *et al.*, 1961), and the purity of each preparation was determined by agarose gel electrophoresis (Nobel, 1968). Each LDL preparation was stored at 4°C and used within 2 weeks of isolation.

Purified LDL was iodinated using an adaptation of the iodine monchloride method described by McFarlane (1958). [$^{125}$I]LDL was not used unless the specific activity fell in the range 200–400 c.p.m. $ng^{-1}$. In the cellular accumulation studies, a working stock of [$^{125}$I]LDL was prepared by dilution with DMEM/1% lipoprotein-deficient fetal calf serum (LPDFCS, Sigma, St Louis, MO, USA). A total of 10 ml was prepared at a concentration of 50 c.p.m. $ng^{-1}$ and 0.5 mg $ml^{-1}$ protein.

Acetylated LDL (Ac-LDL) was prepared by reacting the free amino groups of the lipoprotein with acetic anhydride. This process increases the net negative charge and destroys the ability of the lipoprotein particle to bind to the LDL receptor (Basu *et al.*, 1976). The increase in net negative charge also increases the electrophoretic mobility of the acetylated LDL; therefore, agarose gel electrophoresis (Nobel, 1968) was used to confirm that the LDL had been successfully acetylated. The trinitrobenzenesulphonic acid (TNBS) assay (Habeeb, 1966) indicated that 30% of the free amino groups in the LDL preparation had been modified.

[$^{14}$C]BPD or BPD was equilibrated with LDL, [$^{125}$I]LDL or Ac-LDL by incubation for 30 min at 37°C before addition to the cells or use *in vivo*. This equilibration resulted in the association of all of the BPD with the LDL or Ac-LDL as shown by gel filtration chromatography (data not shown). The concentrations of BPD, LDL and Ac-LDL used in each preparation are reported in the individual experiments.

$R^1 = R^2 = CO_2Me \quad R^3 = (CH_2)_2\,CO_2\,Me$ or $(CH_2)_2CO_2H$

**Figure 1** Structure of benzoporphyrin derivative. R$^3$ represents the hydrolytic site for formation of the mono- and diacid derivatives. BPD monoacid ring A (BPD-MA) was used exclusively in these studies.

*Accumulation of [$^{14}$C]BPD-LDL or [$^{14}$C]BPD-Ac-LDL by cultured fibroblasts* in vitro

Fibroblast cells (1 × $10^5$) were seeded into 60 mm Petri dishes containing 3 ml of DME with 20% fetal calf serum (FCS) on day zero. On day 3, the medium was replaced with fresh medium containing 10% FCS. On day 5 or 6, when the cells were approximately 80% confluent, each dish was washed with 2 ml of PBS and the medium was replaced with 2 ml of DME containing 2.5 mg $ml^{-1}$ LPDFCS. Incubation of the cells in LPDFCS serves to increase the number of LDL receptors per cell (Goldstein *et al.*, 1983). The binding and uptake of the BPD-LDL mixtures was studied after the cells had been incubated for 48 h in medium with LPDFCS.

Prior to each experiment, the medium was removed from the dishes and the cells were washed once with 2 ml of PBS. The PBS was replaced with 1 ml of DME containing the appropriate concentrations of BPD and LDL or Ac-LDL and 1% LPDFCS in the presence or absence of 25-fold excess LDL or Ac-LDL. The cells were incubated in these solutions for 2 h at 37°C. The dishes of cells were then transferred to 4°C and washed three times for 2 min with 4°C PBS containing 2 mg $ml^{-1}$ BSA, followed by two 2 min incubations in 4°C PBS. The cells were then dissolved by exposure to 1 ml of 0.1 M sodium hydroxide for 30 min. One aliquot of the cell lysate (750 µl) was used to determine the amount of [$^{14}$C]BPD that was associated with the cells. Another aliquot (50 µl) was used to determine the amount of cellular protein per dish using the Lowry procedure (Lowry *et al.*, 1961).

*Comparison of [$^{14}$C]BPD-LDL and BPD-[$^{125}$I]LDL accumulation by cultured fibroblasts*

In these experiments, the accumulation of BPD-LDL by normal fibroblasts was studied using both [$^{14}$C]BPD-LDL and BPD-[$^{125}$I]LDL as markers for LDL binding and internalisation. These experiments were performed as described for the *in vitro* accumulation of [$^{14}$C]BPD-LDL except that in this case the BPD was premixed with LDL at a constant ratio of 5 ng BPD per µg of LDL as the LDL concentration was increased (corresponding to a 21:1 molar ratio of BPD-LDL). Parallel experiments were performed with both [$^{14}$C]BPD-LDL mixtures and BPD-[$^{125}$I]LDL mixtures and compared. In the case of the BPD-[$^{125}$I]LDL experiments, the total cellular [$^{125}$I]LDL was measured as indicated for the [$^{14}$C]BPD-LDL mixtures. In addition, the proteolytic hydrolysis of the [$^{125}$I]LDL was measured. LDL that is bound to the LDL receptor is subsequently internalised and delivered to lysosomes, where its protein and cholesteryl ester components are hydrolysed. Hydrolysis of the $^{125}$I-labelled protein leads to secretion of labelled amino acids into the medium, which were distinguished as trichloroacetic acid-soluble material (Goldstein *et al.*, 1974).

*Accumulation of [$^{14}$C]BPD-LDL by cultured M-1 tumour cells*

The experiments with the M-1 cells *in vitro* were performed as described for the accumulation of [$^{14}$C]BPD-LDL in the fibroblast cell lines. Increasing concentrations of BPD were mixed with 10 µl $ml^{-1}$ LDL before addition to these cells. The non-specific binding of [$^{14}$C]BPD-LDL was measured in the presence of a 25-fold excess of native LDL.

*Accumulation of [$^{14}$C]BPD-LDL and [$^{14}$C]BPD-Ac-LDL by tumour tissue* in vivo

The accumulation of BPD into tumours was studied in mature DBA/2J mice bearing the M-1 tumour. [$^{14}$C]BPD (90 µCi $mg^{-1}$) was equilibrated with native or Ac-LDL (2 mg $ml^{-1}$ in Tris-EDTA buffer) by incubating for 30 min at 37°C before intravenous injection into the tail veins of mice. Each mouse received a dose of 4 mg of BPD per kg body weight. They were allowed to eat and drink *ad libitum*.

At 3 h post injection, mice were sacrificed by cervical dislocation under halothane anaesthesia and samples of blood, liver and tumour tissue were removed. Samples were placed in 7 ml vials, minced and the wet weight or volume was determined. The amount of BPD recovered in each tissue was determined as previously described (Richter *et al.*, 1990).

### Results

### *Accumulation of [$^{14}$C]BPD-LDL by cultured fibroblasts*

The kinetics of LDL binding and internalisation by the cell lines used was confirmed by preliminary [$^{125}$I]LDL uptake studies. The measured [$^{125}$I]LDL binding to the normal fibroblasts (GM3348B) was similar to the LDL binding curves published by (Goldstein *et al.* (1974), whereas no specific binding of [$^{125}$I]LDL occurred with the receptor-negative cells.

The ability of these fibroblasts to accumulate BPD-LDL was studied at several concentrations of [$^{14}$C]BPD which had been pre-equilibrated with 10 $\mu$g ml$^{-1}$ LDL. At this concentration, non-specific binding of LDL is usually less than 5–10% of the total binding in the normal cell line (Goldstein *et al.*, 1983), thus differences in the uptake by the two cell lines probably reflect differences in the activity of the LDL receptor. Figure 2 shows the accumulation of BPD in both cell lines. The normal fibroblast cell line displayed concentration-dependent accumulation of [$^{14}$C]BPD-LDL, whereas receptor-negative cells had very little [$^{14}$C]BPD associated with them at any concentration of [$^{14}$C]BPD-LDL. This suggested that the uptake of BPD-LDL by the normal fibroblasts was dependent on LDL receptor-mediated endocytosis.

### *Comparison of [$^{14}$C]BPD-LDL and BPD-[$^{125}$I]LDL accumulation by cultured fibroblasts*

In order to determine the molar ratio of the uptake of BPD and LDL, a comparison of [$^{14}$C]BPD-LDL and BPD-[$^{125}$I]LDL accumulation was performed on the normal fibroblasts. Parallel dishes of cells were incubated with the two mixtures separately for 2 h at 37°C before harvesting the cells. Figure 3 shows the amount of LDL bound and internalised by these cells as determined by the accumulation of [$^{14}$C]BPD-LDL or BPD-[$^{125}$I]LDL. The degradation of LDL was taken into account for measurements of the accumulation of [$^{125}$I]LDL-BPD. BPD accumulated within extrahepatic cells is thought not to be degraded within this time frame (A.M. Richter, 1993, personal communication).

If LDL-associated BPD was endocytosed via the LDL receptor the estimated amount of LDL taken up by the cells would be the same regardless of which component (protein or BPD) was labelled. Figure 3 shows that this was indeed the case. By contrast, if BPD was transferred from the LDL to the plasma membrane of the cells by non-specific processes, the predicted uptake of LDL determined from the [$^{14}$C]BPD-LDL uptake study would be greater than that measured by the uptake of BPD-[$^{125}$I]LDL. Thus, the data in Figure 3 add further support to the hypothesis that LDL-associated BPD is delivered to cells through the LDL receptor pathway. As the concentration of LDL was increased from 10 to 50 $\mu$g ml$^{-1}$, we observed a slight increase in the calculated association of LDL in the presence of the [$^{14}$C]BPD-LDL, however these differences were not significant.

**Figure 3** Comparison of BPD-[$^{125}$I]LDL and [$^{14}$C]BPD-LDL association with GM3348B cells. The LDL (ng $\mu$g$^{-1}$ cell protein) accumulated in the fibroblasts was measured in the presence of [$^{14}$C]BPD-LDL (□) and BPD-[$^{125}$I]LDL (×) as a function of increasing LDL concentration. As the LDL concentration was increased the ratio of BPD to LDL was kept constant at 5 ng of BPD per $\mu$g of LDL. When [$^{14}$C]BPD-LDL was used the ng of LDL associated with the cells was calculated as a function of this ratio in which the BPD and LDL were originally mixed. For every ng of [$^{14}$C]BPD counted with the cells 0.2 $\mu$g of LDL was calculated to be associated. Determinations for each concentration of LDL used were performed in triplicate and the experiments were repeated four times. Therefore, each value shown represents the average of 12 determinations and the error bars represent the standard error of these determinations.

**Figure 2** [$^{14}$C]BPD-LDL accumulation in two fibroblast cell lines. [$^{14}$C]BPD was premixed with 10 $\mu$g ml$^{-1}$ LDL before addition to (■) GM3348B and (×) GM2000E cell lines. Points on the graph represent ng of BPD per $\mu$g of cell protein. Determinations for each concentration of [$^{14}$C]BPD used were performed in duplicate with each experiment being repeated three times. Therefore, each value reported represents the average of six determinations and the error bars represent the standard error of these determinations.

### *The effect of LDL acetylation on [$^{14}$C]BPD-LDL accumulation by cultured fibroblasts*

Since acetylation of LDL abolishes its ability to bind to the LDL receptor (Basu *et al.*, 1976), we investigated the ability of Ac-LDL to deliver BPD to normal fibroblasts. Figure 4a shows that acetylation of LDL decreased the ability of [$^{14}$C]BPD-Ac-LDL to be bound and internalised by these

**Figure 4** Accumulation of [14C]BPD-LDL and [14C]BPD-Ac-LDL in GM3348B cells. In the same series of experiments as in Figure 2, [14C]BPD was premixed with 10 μg ml$^{-1}$ LDL (□) or Ac-LDL (▲) before addition to GM3348B cells (**a**). In **b** a 25-fold excess of either LDL (Δ) or Ac-LDL (O) was added to each [14C]BPD-LDL mixture immediately prior to addition of the solutions to the cells. (■) 10 μg ml$^{-1}$ LDL alone. Points on the graphs represent the ng of BPD recovered per μg of cell protein. Determinations for each concentration of [14C]BPD used were performed in duplicate and the experiments were repeated three times. Therefore, each value plotted represents the average of six determinations and the error bars represent the standard error of these determinations.

cells in comparison with [14C]BPD associated with native LDL. Since very little [14C]BPD-Ac-LDL was accumulated by the fibroblasts, we believe that specific binding of the [14C]BPD-LDL mixture to the LDL receptor accounted for virtually all of the [14C]BPD recovered in these cells when associated with native LDL.

In order to confirm the specificity of this process, we studied the effect of excess LDL or Ac-LDL on [14C]BPD-LDL binding and internalisation by the normal fibroblasts. Figure 4b shows that the accumulation of [14C]BPD-LDL was almost completely inhibited by the addition of 25-fold excess native LDL. Presumably, inhibition was due to competition with the [14C]BPD-LDL for the LDL receptor sites on the cell. By contrast, addition of a 25-fold excess of Ac-LDL had much less effect on the association [14C]BPD-LDL with these cells, since it did not compete for the LDL receptor.

### *[14C]BPD-LDL accumulation by cultured M-1 tumour cells*

All of the experiments with the fibroblasts described above consistently showed that the LDL receptor was responsible for virtually all of the accumulation of BPD-LDL observed. To determine whether this also occurred in cell types which would be treated by PDT, the accumulation of [14C]BPD-LDL was also measured in M-1 tumour cells *in vitro*. In Figure 5, the total accumulation of [14C]BPD-LDL observed was proportional to the [14C]BPD concentration added to these cells. When an excess of LDL was added to the [14C]BPD-LDL mixture, the total accumulation of [14C]BPD-LDL was significantly decreased. Subtraction of the amount associated with the cells in the presence of excess LDL from the total accumulation suggested that the majority (60–70%) of the accumulation was LDL receptor mediated. These results further suggest that the LDL receptor is involved in the uptake of [14C]BPD-LDL by these tumour cells as well as the fibroblasts.

### *Accumulation of [14C]BPD-LDL and [14C]BPD-Ac-LDL mixtures by tumour tissue*

Since the experiments described above clearly indicated that LDL-associated BPD was delivered to cultured cells via the LDL receptor, we extended our studies to investigate whether this mechanism was also involved in the delivery of LDL-associated BPD *in vivo*. Therefore, the biodistribution of BPD equilibrated with either native LDL or Ac-LDL was compared in M-1 tumour-bearing mice. [14C]BPD-LDL and [14C]BPD-Ac-LDL mixtures showed several differences in biodistribution (Table I). However, at the time that the samples were taken, the levels of [14C]BPD-LDL and [14C]BPD-Ac-LDL measured in the blood were not significantly

**Figure 5** [14C]BPD-LDL association with M-1 cells. [14C]BPD was premixed with 10 μg ml$^{-1}$ LDL and added to M-1 tumour cells. The M-1 cells were grown following the method outlined for the human fibroblasts. The association of [14C]BPD with these cells was measured in the absence (□) and presence (■) of 25-fold excess LDL to determine the specific (×) association of [14C]BPD-LDL with these cells. Determinations for each concentration of [14C]BPD used were performed in duplicate and the experiments were performed three times. Therefore, each value plotted represents the average of six determinations and the error bars represent the standard error of these determinations.

**Table I** *In vivo* accumulation of [$^{14}$C]BPD-LDL and [$^{14}$C]BPD-Ac-LDL

| Tissue | Recovery of [$^{14}$C]BPD (ng mg$^{-1}$ tissue) LDL | Ac-LDL |
|---|---|---|
| Tumour | 1.17 ± 0.14 | 0.70 ± 0.08* |
| Liver | 7.40 ± 1.27 | 3.26 ± 0.39** |
| Blood | 1.86 ± 0.09 | 1.52 ± 0.28 |

[$^{14}$C]BPD (90 μCi mg$^{-1}$) was equilibrated with native or Ac-LDL (2 mg ml$^{-1}$ in Tris-EDTA buffer) before intravenous injection into M-1 tumour-bearing DBA/2J mice. Each mouse received a dose of 4 mg of BPD per kg body weight. At 3 h post injection mice were sacrificed and tissue samples were excised. The [$^{14}$C]BPD (ng mg$^{-1}$) recovered in each tissue was determined. The values displayed represent the mean of samples from five mice and the standard error of these samples. *$P<0.01$, **$P<0.05$.

different (1.9 ± 0.1 ng mg$^{-1}$ and 1.52 ± 0.28 ng mg$^{-1}$ respectively, $P>0.4$). Although considerable proportions of each preparation were recovered in the liver, this was markedly ($P<0.05$) higher in the presence of native LDL (7.4 ± 1.27 ng mg$^{-1}$ for [$^{14}$C]BPD-LDL; 3.26 ± 0.40 ng mg$^{-1}$ for [$^{14}$C]BPD-Ac-LDL). The [$^{14}$C]BPD deposited per mg of tumour tissue in the presence of both lipoprotein preparations suggested that LDL receptor-mediated uptake of the [$^{14}$C]BPD-LDL complexes (1.17 ± 0.14) significantly ($P<0.01$) enhanced the accumulation of the drug in the tumour compared with that of the [$^{14}$C]BPD-Ac-LDL complexes (0.70 ± 0.08).

### Discussion

In these studies, three different strategies were used to elucidate the role of the LDL receptor in the cellular accumulation of BPD-LDL mixtures. Fibroblast cell lines were chosen for these studies because they have been used extensively for characterisation of the LDL receptor (Goldstein *et al.*, 1974, 1983). Marked differences were observed between [$^{14}$C]BPD-LDL accumulation by normal and LDL receptor-negative fibroblasts, suggesting that the LDL receptor played a major role. Little difference was observed between the accumulation of [$^{14}$C]BPD-LDL and BPD-[$^{125}$I]LDL in normal fibroblasts, indicating that BPD did not dissociate from the LDL to the cells during the incubations. Finally, chemical acetylation of LDL allowed us to determine directly whether the LDL receptor was involved in the accumulation of [$^{14}$C]BPD-LDL by the cells. The difference observed between the association of [$^{14}$C]BPD-LDL and [$^{14}$C]BPD-Ac-LDL with the normal fibroblast cell line indicated that photosensitiser accumulation was due almost exclusively to specific binding and internalisation via the LDL receptor. Experiments performed using excess LDL or acetyl-LDL confirmed these observations.

In previous *in vitro* studies, Candide *et al.* (1980) showed that Photofrin II was taken up by cultured human fibroblasts more efficiently when mixed with LDL than with HDL or albumin. However, the LDL-Photofrin II uptake was greater than expected when the cells were grown in lipoprotein-containing medium. Under these conditions the cellular LDL receptor expression should be low. These investigators concluded that a non-specific LDL-Photofrin II uptake was involved in addition to the LDL receptor-mediated pathway. They later proposed that non-specific exchange of porphyrins between LDL and the plasma membrane occurs (Mazière *et al.*, 1990). In contrast, we observed very little accumulation of [$^{14}$C]BPD-LDL by LDL receptor-negative fibroblasts. In a separate experiment with the normal fibroblasts, equivalent accumulation of [$^{14}$C]BPD-LDL and BPD-[$^{125}$I]LDL was observed. Both these experiments suggest that the BPD did not dissociate from the LDL and partition into the plasma membrane of these cells. The behaviour of LDL-associated BPD or Photofrin II may be related to the relative affinity of these photosensitisers for the LDL. This has been reported to be a function of the hydrophobicity of the photosensitiser (Jori, 1989; Kongshaug *et al.*, 1989). Thus, the nature of the association of BPD and Photofrin II with LDL may differ, especially since Photofrin II consists of an aggregated mixture of compounds (Dougherty *et al.*, 1987; Kessel *et al.*, 1987), most of which are not highly hydrophobic. Differences in photosensitisers, such as this, may be partially responsible for the conflicting LDL delivery results which had been previously reported (Korbelik *et al.*, 1990).

*In vivo* studies have demonstrated that the association of several different photosensitisers with LDL can enhance their delivery to malignant tissues (Jori *et al.*, 1984; Zhou *et al.*, 1988; Mazière *et al.*, 1991). Results of *in vivo* studies on the distribution, elimination and cytotoxic effects of photosensitisers in experimental tumours have been explained by eluding to the mechanism of LDL receptor-mediated endocytosis of photosensitisers, without experimental confirmation of the process (Kessel, 1986; Mazière *et al.*, 1990; Allison *et al.*, 1991; Richter *et al.*, 1991). Our *in vivo* studies have extended the observations of LDL receptor-mediated accumulation of BPD *in vitro* to a credible mechanism for the delivery of BPD to tumour tissue *in vivo*.

We compared the ability of native LDL and acetylated LDL to deliver BPD to M-1 tumours *in vivo*. Modification of 15% of the peptidyl lysines of LDL abrogates binding to the LDL receptor (Goldstein *et al.*, 1979; Haberland *et al.*, 1984; Via *et al.*, 1992) but acetylation of >29% is necessary for recognition of Ac-LDL by the scavenger receptor which recognises modified LDL (Goldstein *et al.*, 1979; Via *et al.*, 1992). This receptor is active on macrophage and endothelial cells, both of which might also be involved in BPD accumulation in the M-1 tumour. The inability of Ac-LDL to deliver BPD to the fibroblasts in our *in vitro* experiments suggests that the acetylated LDL used was no longer binding to the LDL receptor. Normally, recognition of Ac-LDL by the scavenger receptor leads to very rapid clearance from the blood via the Kupffer cells of the liver (Goldstein *et al.*, 1979; Van Berkel *et al.*, 1991). The markedly higher accumulation of [$^{14}$C]BPD-LDL in the liver and the similar blood levels of [$^{14}$C]BPD-LDL and [$^{14}$C]BPD-Ac-LDL observed 3 h post administration *in vivo* suggest that our Ac-LDL preparations were not recognised by the scavenger receptor. The TNBS assay performed on the acetylated LDL indicated that 30% of the lysines were modified, which might not have been sufficient for recognition by the scavenger receptor. Thus, the difference observed in the tumour accumulation of the two different BPD-LDL preparations was probably due exclusively to LDL receptor-mediated accumulation of the native LDL-associated BPD.

Our previous studies have suggested that the association of BPD with LDL increases access of the photosensitiser to the tumour cells within the tumour tissue (Allison *et al.*, 1991). Similarly, Zhou *et al.* (1988) have reported that the association of Zn phthalocyanine with LDL increases the damage to tumour cells (as opposed to tumour vasculature) upon exposure to light. In the context of our present *in vivo* study, it seems likely that the enhanced uptake of native LDL by tumour cells accounts for the difference between LDL and Ac-LDL with respect to BPD delivery to the tumour. However, our measurements of [$^{14}$C]BPD-LDL in the tumour tissue did not distinguish between intra- and extracellular tissue uptake. The accumulation of a portion of [$^{14}$C]BPD-LDL and [$^{14}$C]BPD-Ac-LDL in the tumour may be extracellular. Very little is currently known about the interaction of photosensitisers with extracellular matrix components or the effects lipoproteins may have on these interactions.

The different experimental strategies described in this paper consistently show that the LDL receptor is responsible for virtually all of the accumulation of LDL-associated BPD *in vitro*. Extension of these studies to tumour-bearing mice indicates that LDL receptor-mediated endocytosis also makes a major contribution to the delivery of this photosensitiser *in vivo*. Taken together, these studies suggest that LDL receptor-mediated endocytosis contributes substantially to the selective accumulation of BPD (and possibly other hydrophobic photosensitisers) into malignant tissues. Characterisation and utilisation of this delivery system may provide for an improvement in PDT in clinical practice.

This work was supported in part by grants from the BC Heart Foundation and the Medical Research Council of Canada. Beth A. Allison was supported by a scholarship from the Science Council of British Columbia and a post-doctoral fellowship from the Natural Science and Engineering Council of Canada. We would like to thank Quadra Logic Technologies and Dr D. Dolphin's laboratory at UBC for the generous donation of BPD and radiolabelled BPD. We would also like to acknowledge Dr U. Steinbrecher for his thought-provoking discussions and experimental suggestions. Excellent technical assistance was provided by E. Donnachie.

**Abbreviations:** PDT, photodynamic therapy; BPD, benzoporphyrin derivative; LDL, low-density lipoprotein; Ac-LDL, acetylated low-density lipoprotein; DMEM, Dulbecco's modified Eagle medium; FCS, fetal calf serum; LPDFCS, lipoprotein-deficient fetal calf serum; PBS, phosphate-buffered saline.

### References

ALLISON, B.A., PRITCHARD, P.H., RICHTER, A.M. & LEVY, J.G. (1990). The plasma distribution of benzoporphyrin derivative and the effects of plasma lipoproteins on its biodistribution. *Photochem. Photobiol.*, **52**, 501–507.

ALLISON, B.A., WATERFIELD, E., RICHTER, A.M. & LEVY, J.G. (1991). The effects of plasma lipoproteins on *in vitro* tumour cell killing and *in vivo* tumour photosensitization with benzoporphyrin derivative. *Photochem. Photobiol.*, **54**, 709–715.

ATTIE, A.D., PITTMAN, R.C. & STEINBERG, D. (1982). Hepatic catabolism of low density lipoprotein: mechanisms and metabolic consequences. *Hepatology*, **2**, 269–281.

BAREL, A., JORI, G., PERIN, F., PAGNAN, A. & BIFFANTI, S. (1986). Role of high-, low- and very low density lipoproteins in the transport and tumor-delivery of hematoporphyrin *in vivo*. Cancer Lett., **32**, 145–150.

BASU, S.K., GOLDSTEIN, J.L., ANDERSON, R.G.H. & BROWN, M.S. (1976). Degradation of cationized LDL and regulation of cholesterol metabolism in homozygous familial hypercholesterolemia. *Proc. Natl Acad. Sci. USA*, **73**, 3178–3182.

CANDIDE, C., MORLIÈRE, P., MAZIÈRE, J.C., GOLDSTEIN, S., SANTUS, R., DUBERTRET, L., REYFTMANN, J.P. & POLONOVSKI, J. (1986). *In vitro* interaction of the photoactive anticancer porphyrin derivative photofrin II with low density lipoprotein, and its delivery to cultured human fibroblasts. *FEBS Lett.*, **207**, 133–138.

DOUGHERTY, T.J. (1987). Studies on the structure of porphyrins contained in Photofrin II. *Photochem. Photobiol.*, **46**, 569–573.

DOUGHERTY, T.J., COOPER, M.T. & MANG, T.S. (1990). Cutaneous phototoxic occurrences in patients receiving Photofrin. *Laser Surg. Med.*, **10**, 485–488.

GAL, D., MCDONALD, P.C., PORTER, J.C. & SIMPSON, E.R. (1981). Cholesterol metabolism in cancer cells in monolayer culture. III. Low density lipoprotein metabolism. *Int. J. Cancer*, **28**, 315–319.

GOLDSTEIN, J.L. & BROWN, M.S. (1974). Binding and degradation of low density lipoproteins by cultured human fibroblasts. *J. Biol. Chem.*, **249**, 5153–5162.

GOLDSTEIN, J.L., HO, Y.K., BASU, S.K. & BROWN, M.S. (1979). Binding site on macrophages that mediates uptake and degradation of acetylated low density lipoproteins, producing massive cholesterol deposition. *Proc. Natl Acad. Sci. USA*, **76**, 333–337.

GOLDSTEIN, J.L., BASU, S.K. & BROWN, M.S. (1983). Receptor-mediated endocytosis of low-density lipoprotein in cultured cells. *Methods Enzymol.*, **98**, 241–260.

GOMER, C.J. (1989). Photodynamic therapy in the treatment of malignancies. *Semin. Hematol.*, **26**, 27–34.

GOTTO, A.M., POWNALL, H.J. & HAVEL, R.J. (1986). Introduction to the plasma lipoproteins. *Methods Enzymol.*, **128**, 3–41.

HABEEB, A.F.S.A. (1966). Determination of free amino groups in proteins by trinitrobenzenesulfonic acid. *Anal. Biochem.*, **14**, 328–336.

HABERLAND, M.E., OLCH, C.L. & FOGELMAN, A.M. (1984). Role of lysines in mediating interaction of modified low density lipoproteins with the scavenger receptor of human monocyte macrophages. *J. Biol. Chem.*, **259**, 11305–11311.

HAVEL, R.J., EDER, H.A. & BRAGDON, J.H. (1955). Distribution and chemical composition of ultracentrifugally separated lipoproteins in human serum. *J. Clin. Invest.*, **34**, 1345–1353.

HENDERSON, B.W. & DOUGHERTY, T.J. (1992). How does photodynamic therapy work? *Photochem. Photobiol.*, **55**, 145–157.

HO, Y.K., SMITH, G.R., BROWN, M.S. & GOLDSTEIN, J.L. (1979). Low density lipoprotein (LDL) receptor activity in human acute myelogenous leukaemia cells. *Blood*, **56**, 1099–1114.

JORI, G. (1989). *In vivo* transport and pharmacokinetic behaviour of tumour photosensitizers. *Ciba Foundation Symposium*, **146**, 78–94.

JORI, G., REDDI, E., SALVATO, B., PAGNAN, A. & ZIRON, L. (1984). Evidence for a major role of plasma lipoproteins as hematoporphyrin carries *in vivo*. *Cancer Lett.*, **24**, 291–297.

KESSEL, D. (1986). Porphyrin-lipoprotein association as a factor in porphyrin localization. *Cancer Lett.*, **33**, 183–188.

KESSEL, D., THOMPSON, P., MUSSELMAN, B. & CHANG, C.K. (1987). Chemistry of hematoporphyrin-derived photosensitizers. *Photochem. Photobiol.*, **46**, 563–568.

KONGSHAUG, M., MOAN, J. & BROWN, S.B. (1989). The distribution of porphyrins with different tumour localising ability among human plasma proteins. *Br. J. Cancer*, **59**, 184–188.

KORBELIK, M., HUNG, J., LAM, S. & PALCIC, B. (1990). The effects of low density lipoprotein on uptake of Photofrin II. *Photochem. Photobiol.*, **51**, 191–196.

LOWRY, O.H., ROSEBROUGH, M.J., FARR, A.L. & RANDALL, R.J. (1961). Protein measurement with folin-phenol reagent. *J. Biol. Chem.*, **193**, 265–275.

LUNDBERG, B. (1991). Techniques for complexing pharamcological agents to lipoproteins and lipid emulsions. In *Lipoproteins as Carriers of Pharmacological Agents*, Shaw, J. M. (ed.), p. 99. Marcel Dekker: New York.

MANYAK, M.J., RUSSO, A., SMITH, P.D. & GLATSTEIN, E. (1988). Photodynamic therapy. *J. Clin. Oncol.*, **6**, 380–391.

MAZIÈRE, J.C., SANTUS, R., MORLIÈRE, P., REYFTMANN, J.P., CANDIDE, C., MORA, L., SALMON, S., MAZIÈRE, GATT, S. & DUBERTRET, L. (1990). Cellular uptake and photosensitizing properties of anticancer porphyrins in cell membranes and low and high density lipoproteins. *J. Photochem. Photobiol. B: Biol.*, **6**, 61–68.

MAZIÈRE, J.C., MORLIÈRE, P. & SANTUS, R. (1991). The role of the low density lipoprotein pathway in the delivery of lipophilic photosensitizers in the photodynamic therapy of tumours. *J. Photochem. Photobiol.*, **8**, 351–360.

MCFARLANE, A.S. (1958). Efficient trace-labelling of proteins with iodine. *Nature*, **182**, 53.

MOAN, J. & BERG, K. (1992). Photochemotherapy of cancer: experimental research. *Photochem. Photobiol.*, **55**, 931–948.

NOBEL, R.P. (1968). Electrophoretic separation of plasma lipoproteins in agarose gel. *J. Lipid Res.*, **9**, 693–700.

NORATA, G., CANTI, G., RICCI, L., NICOLIN, A., TREZZI, E. & CATAPONA, A.L. (1984). *In vivo* assimilation of low density lipoproteins by a fibrosarcoma tumour line in mice. *Cancer Lett.*, **25**, 203–208.

PANKA, J.S., MORGAN, A.R. & DOLPHIN, D. (1986). Diels-Alder reactions of protoporphyrin IX dimethylester with electron-deficient alkynes. *J. Org. Chem.*, **51**, 1094–1100.

PATERSON, H.I. & APPERGREN, K.L. (1973). Experimental studies on the uptake and retention of labelled proteins in a rat tumor. *Eur. J. Cancer*, **9**, 109–116.

RICHTER, A.M., KELLY, B., CHOW, J., LIU, D., TOWERS, G.H., DOLPHIN, D. & LEVY, J.G. (1987). Preliminary studies of a more effective phototoxic agent that hematoporphyrin. *J. Natl Cancer Inst.*, **79**, 1327–1332.

RICHTER, A.M., CERRUTI-SOLA, S., STERNBERG, E.D., DOLPHIN, D. & LEVY, J.G. (1990). Biodistribution of tritiated benzoporphyrin derivative ($^3$H-BPD-MA), a new potent photosensitizer, in normal and tumour-bearing mice. *J. Photochem. Photobiol., B, Biology*, **5**, 231–244.

RICHTER, A.M., WATERFIELD, E., JAIN, A.K., ALLISON, B., STERNBERG, E.D., DOLPHIN, D. & LEVY, J.G. (1991). Photosensitising potency of structural analogues of benzoporphyrin derivative (BPD) in a mouse tumour model. *Br. J. Cancer*, **63**, 87-93.

VAN BERKEL, T.J.C., KRUIJT, J.K., DE SMIDT, P.C. & BIJSTERBOSCH, M.K. (1991). Receptor-dependent targeting of lipoproteins. In *Lipoproteins as Carriers of Pharmacological Agents*, Shaw, J. M. (ed.), pp. 247-248. Marcel Dekker: New York.

VIA, D.P., KEMPER, E.S., PONS, L., FANSLOW, A.E., VIGNALE, S., SMITH, L.C. & GOTTO Jr, A.M. (1992). Mouse macrophage receptor for acetylated low density lipoprotein: demonstration of a fully functional subunit in the membrane and with purified receptor. *Proc. Natl Acad. Sci. USA*, **89**, 6780-6784.

VITOLS, S., GAHRTON, G., ÖST, Ä. & PETERSON, C. (1984). Elevated low density lipoprotein receptor activity in leukaemic cells with monocytic differentiation. *Blood*, **63**, 1186-1193.

WEISHAUPT, K., GOMER, C.G. & DOUGHERTY, T.J. (1976). Identification of singlet oxygen as the cytotoxic agent in photoinactivation of a murine tumour. *Cancer Res.*, **36**, 2326-2329.

ZHOU, C., MILANESI, C. & JORI, G. (1988). An ultrastructural comparative evaluation of tumours photosensitized by porphyrins administered in aqueous solution, bound to liposomes or to lipoproteins. *Photochem. Photobiol.*, **48**, 487-492.