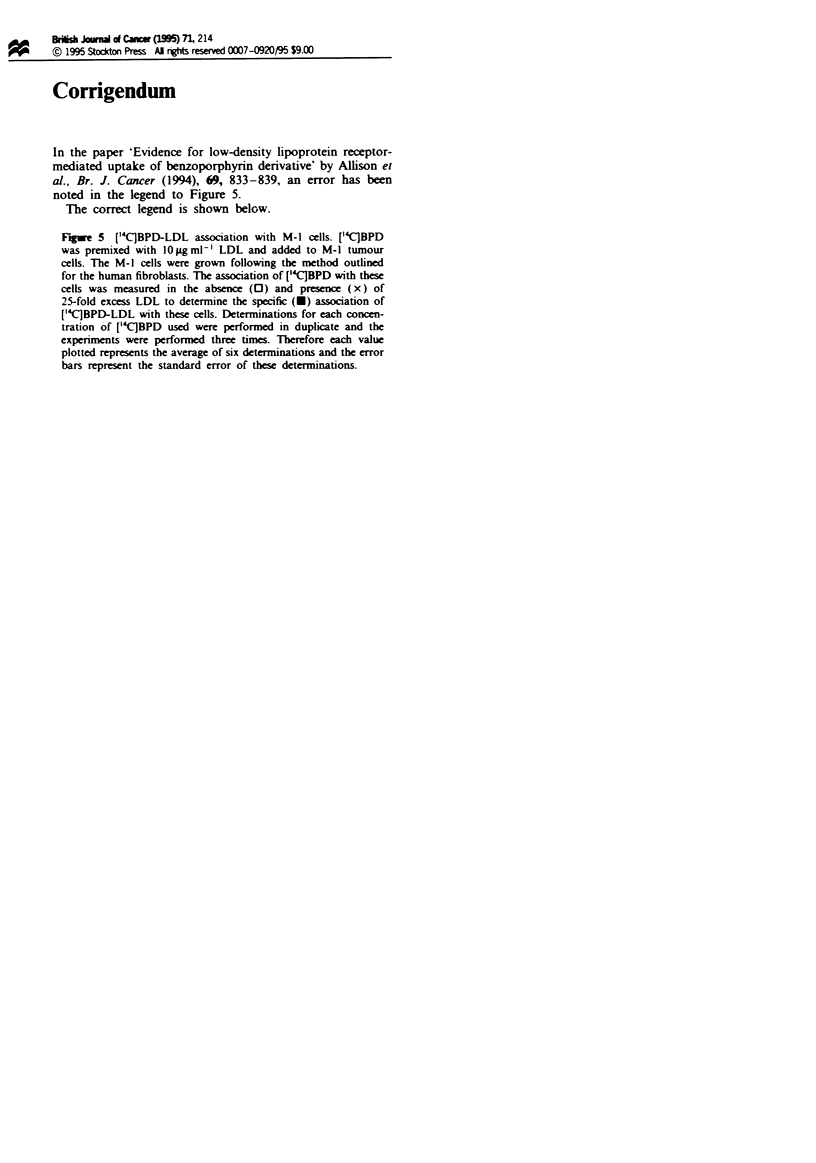# Corrigendum

**Published:** 1995-01

**Authors:** 


British Journal of Cancer (1995) 71, 214

© 1995 Stockton Press All rights reserved 0007-0920/95 $9.00

### Corrigendum

In the paper 'Evidence for low-density lipoprotein receptor-mediated uptake of benzoporphyrin derivative' by Allison *et al.*, *Br. J. Cancer* (1994), **69**, 833–839, an error has been noted in the legend to Figure 5.

The correct legend is shown below.

**Figure 5** [$^{14}$C]BPD-LDL association with M-1 cells. [$^{14}$C]BPD was premixed with 10 $\mu$g ml$^{-1}$ LDL and added to M-1 tumour cells. The M-1 cells were grown following the method outlined for the human fibroblasts. The association of [$^{14}$C]BPD with these cells was measured in the absence (□) and presence (×) of 25-fold excess LDL to determine the specific (■) association of [$^{14}$C]BPD-LDL with these cells. Determinations for each concentration of [$^{14}$C]BPD used were performed in duplicate and the experiments were performed three times. Therefore each value plotted represents the average of six determinations and the error bars represent the standard error of these determinations.